# Gender Differences in a *Drosophila* Transcriptomic Model of Chronic Pentylenetetrazole Induced Behavioral Deficit

**DOI:** 10.1371/journal.pone.0008136

**Published:** 2009-12-02

**Authors:** Abhay Sharma, Farhan Mohammad, Priyanka Singh

**Affiliations:** Institute of Genomics and Integrative Biology, Council of Scientific and Industrial Research, Delhi University Campus, Delhi, India; Deutsches Krebsforschungszentrum, Germany

## Abstract

A male *Drosophila* model of locomotor deficit induced by chronic pentylenetetrazole (PTZ), a proconvulsant used to model epileptogenesis in rodents, has recently been described. Antiepileptic drugs (AEDs) ameliorate development of this behavioral abnormality. Time-series of microarray profiling of heads of male flies treated with PTZ has shown epileptogenesis-like transcriptomic perturbation in the fly model. Gender differences are known to exist in neurological and psychiatric conditions including epileptogenesis. We describe here the effects of chronic PTZ in *Drosophila* females, and compare the results with the male model. As in males, chronic PTZ was found to cause a decreased climbing speed in females. In males, overrepresentation of Wnt, MAPK, TGF-beta, JAK-STAT, Cell communication, and Dorso-Ventral axis formation pathways in downregulated genes was previously described. Of these, female genes showed enrichment only for Dorso-Ventral axis formation. Surprisingly, the ribosomal pathway was uniquely overrepresented in genes downregulated in females. Gender differences thus exist in the *Drosophila* model. Gender neutral, the developmental pathway Dorso-Ventral axis formation may be considered as the candidate causal pathway in chronic pentylenetetrazole induced behavioral deficit. Prior evidence of developmental mechanisms in epileptogenesis may support potential usefulness of the fly model. Given this, gender specific pathways identified here may provide a lead for further understanding brain dimorphism in neuropsychiatric disorders.

## Introduction

The prevalence and course of various neurological and psychiatric disorders are known to differ between the sexes [*1*], [*2*]. Sexually dimorphic CNS structure and function are known in animals as well [*3*]. The gender-specific differences have been observed at various levels including gene expression, metabolism and cell division [*4*], [5**]. For example, sex-specific differences in brain metabolism have been observed in epileptic patients [*4*]. Similarly, seizures have been found to cause gender-specific effect on cell proliferation and survival in rats [*5*]. Differences in patterns of gene expression have been suggested as one of the contributing factor in sexual dimorphism in neuropsychiatric disorders [*6*].

A male *Drosophila* model of locomotor deficit induced by chronic pentylenetetrazole (PTZ), a proconvulsant used to model epileptogenesis in rodents, has recently been described [*7*]. Antiepileptic drugs (AEDs) have been found to ameliorate development of this behavioral abnormality. Time-series of microarray profiling of heads of male flies treated with PTZ has shown that gene expression changes in the fly model resemble, to some extent, that in epileptogenesis [*7*]. It has been argued that amenability of *Drosophila* to systems level modeling may tempt further dissection of the fly PTZ model to examine its potential usefulness in understanding epileptogenesis-like plasticity and in unraveling mechanisms of long-term action of AEDs relevant in neuropsychiatric conditions [*7*]. Given the potential of the fly model in understanding sex differences in pathological condition, we developed the female counterpart of the male model and examined gender differences, if any. We describe here the effects of chronic PTZ in *Drosophila* females, and compare the results with the male model. We do observe gender differences in the fly model at transcriptomic level.

## Results

Chronic treatment with PTZ for seven days was earlier described to result in a decreased climbing speed in males [*7*]. We similarly treated females and measured the climbing speed. Whereas the control NF treated flies (*n* = 24) climbed with a speed of 1.23 cm/sec, PTZ treated ones (*n* = 24) showed a climbing speed of 0.86 cm/sec. Pair-wise Student's *t*-test, two-tailed, heteroscedastic, showed the gender difference in climbing speed as significant (*p* = 0.001). Females were thus found comparable to males in terms of behavioral effect of chronic PTZ.

Time-series of microarray expression profiles of heads of male flies at 0 hr and after PTZ treatment for 12 hrs, two days and seven days have previously been described [*7*]. Numbers of analyzable genes in SAM (Significant Analysis of Microarrays) in male microarrays at these time-points were 4369, 4637, 5259 and 4297, in that order [*7*]. At 0 hr, no gene was detected as differentially expressed below 96% FDR (false Discovery rate). At 12 hrs, 2^nd^ day and 7^th^ day time-points, 23, 2439 and 265 genes were found to be downregulated at 22.76%, 13.74% and 23.03% FDR, in that order [*7*]. No upregulated gene was detected at these FDRs [*7*]. Given small number of genes at 12 hrs, these FDRs were considered as the best compromise between uniformity across time points and acceptability in terms of incorporating false positives [*7*]. To compare with males, we generated microarray gene expression profiles of heads of female flies after 12 hrs, 2 days and 7 days of PTZ treatment using the same materials and methods. Analyzable genes in SAM were found to be 5307, 5346 and 6995, in that order. At 12 hrs, 2^nd^ day and 7^th^ day time-points, 1, 3 and 1 genes were upregulated, and 1, 68 and 800 genes were downregulated at 0%, 16% and 19% FDR, in that order (**Table S1**). As the next FDR jumped to 43% at 12 hrs time point, we considered the above FDRs as comparable to male profiles.

**Figure 1:**
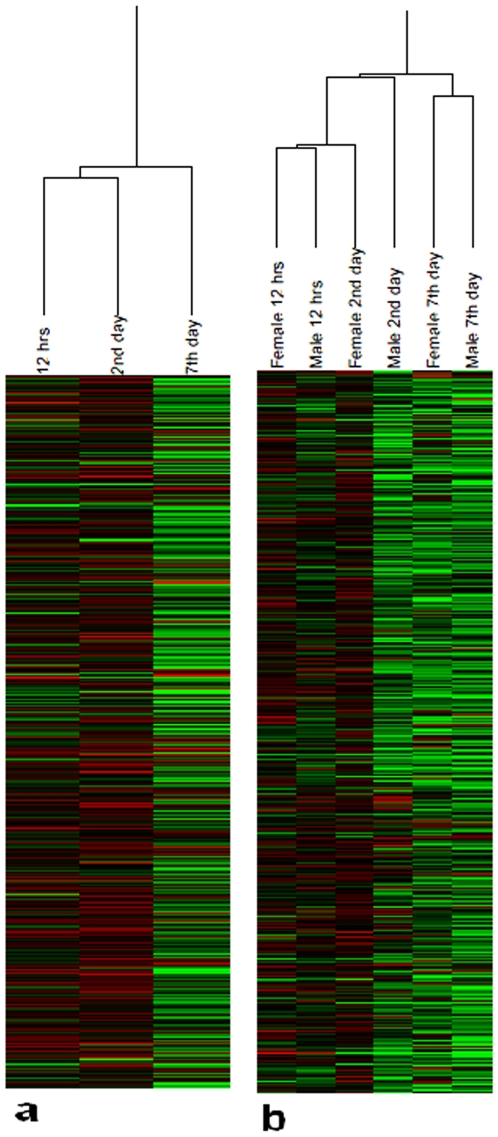
Time-series of microarray gene expression profiles. Profiles were generated from heads of PTZ treated flies. Hierarchical clustering of female profiles alone (a), and both male and female profiles together (b). Male microarrays were described previously [*7*]. City Block similarity metric and average linkage methods were used for clustering of arrays. Each profile represents mean of normalized log_2_ ratio (635/532) of four biological replicates with balanced dye-swaps. The cluster was generated using Acuity 4.0.

Earlier, clustering of male microarrays was found to be consistent with the time-series [*7*]. We clustered the female microarrays and examined their consistency. Like males, female microarrays also clustered according to the time-series (**Fig. 1a**). To examine possible gross level sex differences, we clustered both male and female time-series together. Except 2^nd^ day time-point where some discrepancy was noted, males and females were found to be similar in their chronological response to PTZ (**Fig. 1b**). As described above, PTZ's overall effect on gene expression in females, like males, was inhibitory (**Table S1**). Thus, we henceforth compared genes downregulated in males and females.

**Figure 2:**
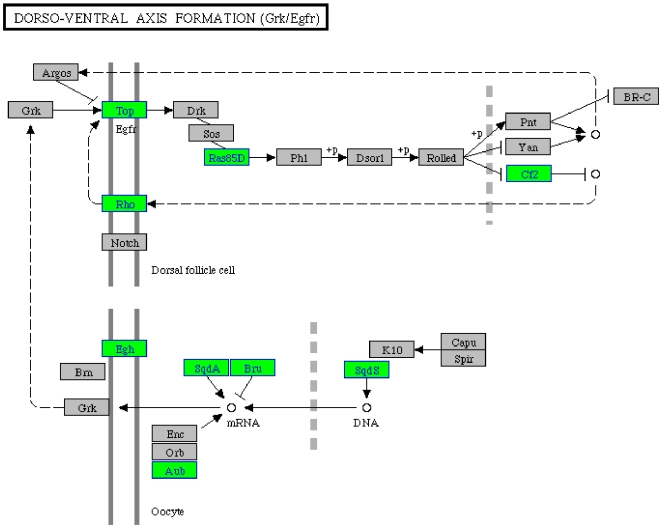
Downregulation of genes related to Dorso-ventral axis formation pathway. Green boxes indicate genes downregulated in females after chronic PTZ. Differentially expressed genes in all the three time-points combined were used for pathway mapping. Dorso-ventral axis formation pathway was previously found to be enriched in males also [*7*].

**Figure 3:**
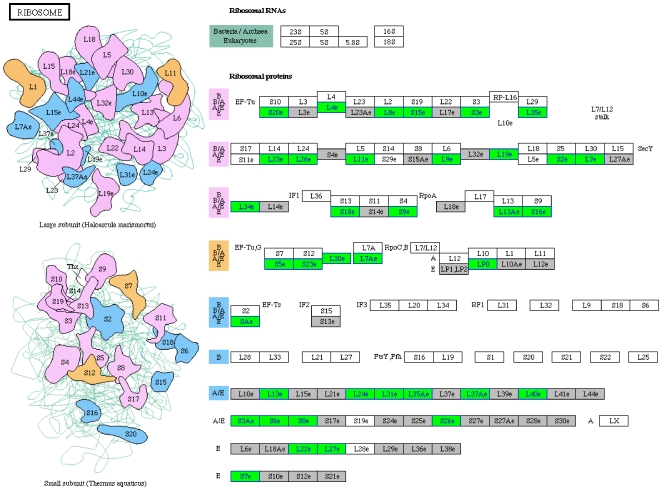
Downregulation of genes related to the ribosomal pathway. Green boxes indicate genes downregulated in females after chronic PTZ. Differentially expressed genes in all the three time-points combined were used for pathway mapping. The ribosomal pathway was previously not found to be enriched in males [*7*]. Grey and white boxes indicate presence and absence of *Drosophila melanogaster* genes in the pathway, respectively.

We identified overrepresented pathways in genes downregulated in females at all three time-points combined (**Table 1**). In males, overrepresentation of Wnt, MAPK, TGF-beta, JAK-STAT, Cell communication, and Dorso-Ventral axis formation pathways in downregulated genes has previously been described [*7*]. Of these, female genes showed enrichment only for Dorso-Ventral axis formation (**Fig. 2**). Further, it was remarkable to note uniquely significant overrepresentation of the ribosomal pathway in females (**Fig. 3**).

**Table 1:** KEGG pathways enriched in genes downregulated in females after chronic PTZ.

Term	p-Value	Benjamini adjusted p-Value
Ribosome	2.60E-16	2.80E-14
Dorso-ventral axis formation	3.90E-02	8.20E-01
Pyruvate metabolism	4.70E-02	7.80E-01

## Discussion

Our results show that gender differences do exist in the *Drosophila* model at transcriptomic level. Whereas Dorso-Ventral axis formation pathway has been found to be common in both sexes, other pathways showed gender bias. Considering similar behavioral effect of PTZ, the gender neutral Dorso-Ventral axis formation may be considered as the pathway causally associated with development of behavioral deficit caused by the chemoconvulsant. Our unbiased analysis thus led us to identify a developmental mechanism in the fly model. It is interesting to note here that gene expression studies have previously implicated developmental mechanisms in epileptogenesis [*8*]. This underscores the relevance of the fly model in disease genomics. The gender specific pathways that we have identified may provide a lead for understanding brain dimorphism. Known translational control of long-lasting brain plasticity [*9]*, [10**], for example, is notable in the context of enrichment of the ribosomal pathway genes in the female fly model. Further, coregulation of ribosomal and energy metabolic pathways have recently been noted in the mechanism of AED action in the male fly model (unpublished). In the female model presented here, we do observe downregulation of pyruvate metabolism along with the ribosomal pathway. This is tempting to speculate that female-specific pathways reflect underlying recovery processes. The *Drosophila* systems model seems to offer a unique opportunity to unravel the gender differences in neuropsychiatric disorders in cellular and molecular terms.

## Materials and Methods

Previously described [*7*] methods were used. In brief, 3–4 days old virgin females were treated with 8 mg/ml of PTZ for varying length of time. Flies treated with normal food (NF) were used as controls. An indigenously developed semi-manual method, validated by Dynamic Image Analysis System (DIAS v. 3.4.2, Soll Technologies), was used to measure climbing speed. In this semi-manual assay, each fly was first familiarized in a vertically placed glass column for 90 seconds and then startle induced climbing activity was recorded using a “dot/comma” method. In “dot/comma” recording, the locomotor activity of a fly was recorded by keep pressing the dot key or the comma key of a personal computer, to record a climbing or a resting fly, in that order. Using the cursor speed, the dots and commas were accordingly transformed in the activity and rest period. Climbing speed was calculated using the following formula, *s* = *h*/*t*, where *s* = climbing speed, *h* = height climbed in cm, and *t* = activity period in sec.

Total cellular RNA was isolated from fly heads belonging to four biological replicates. Microarray -cDNA Synthesis Kit, -Target Purification Kit, and -RNA Target Synthesis Kit (Roche) were used to generate labeled antisense RNA. Starting with 10 µg of total cellular RNA, Eberwine method (kits from Roche) was used to generate cDNA and thereafter Cy^3^ and Cy^5^ (Amersham) labeled antisense RNA. The Cy^3^ and Cy^5^ labeled aRNAs (control and treated) were pooled together and precipitated, washed, air-dried, and dissolved in 18 MΩ RNAase free water. A total of 12 microarrays (12 Kv1, CDMC) were hybridized, four each for 12 hrs, 2^nd^ day and 7^th^ day of PTZ treatment. Out of four, two slides were dye-swaps. Slides were scanned at 10 µm resolution using GenePix 4000A Microarray Scanner (Molecular Devices) and the images preprocessed and quantified using Gene Pix Pro 6.0 (Molecular Devices). All microarray data reported in the manuscript is described in accordance with MIAME guidelines. The full microarray data set has been deposited in the Gene Expression Omnibus (http://www.ncbi.nlm.nih.gov/geo/) under accession series GSE10852. Ratio based data normalization and selection of features were performed using Acuity 4.0 (Molecular Devices). All Spots with raw intensity less then 100U and less then twice the average background was ignored during normalization. Normalized data was filtered for the selection of features before further analysis. Only those spots were selected which contained only a small percentage (<3) of saturated pixels, were not flagged bad or found absent (flags>0), had relatively uniform intensity and uniform background [Rgn R2 (635/532)>0.6] and were detectable above background (SNR>3). Analyzable spots in at least three of four biological replicates performed were retrieved for downstream analysis using SAM (v.3.0, Excel Add-In) [*11*], under the conditions of one class response and 100 permutations. DAVID [*12*] was used for pathway enrichment analysis (http://david.abcc.ncifcrf.gov/home.jsp). KEGG pathway (http://www.genome.jp/kegg/tool/color_pathway.html) was used for mapping of fly genes.

## Supporting Information

Table S1 Differentially expressed genes in females at 12 hrs, 2nd day and 7th day of PTZ treatment. List of up- and down-regulated genes in heads of female flies treated with PTZ for 12 hrs, two days, and seven days.
(0.12 MB XLS)
